# Improving dataset transparency in dermatologic Artificial Intelligence using a dataset nutrition label

**DOI:** 10.1038/s41746-025-02125-9

**Published:** 2025-11-05

**Authors:** Yingjoy Li, Matthew Taylor, Kasia S. Chmielinski, Allan C. Halpern, Roxana Daneshjou, Jenna C. Lester, Veronica Rotemberg

**Affiliations:** 1 Dermatology Service, Department of Medicine, Memorial Sloan Kettering Cancer Center, New York, NY USA; 2 The Data Nutrition Project, Jersey City, NJ USA; 3 Berkman Klein Center for Internet & Society, Harvard University, Cambridge, MA USA; 4 Department of Dermatology, Stanford School of Medicine, Stanford, CA USA; 5 Department of Dermatology, University of California, San Francisco School of Medicine, San Francisco, CA USA

**Keywords:** Computational biology and bioinformatics, Diseases, Health care, Mathematics and computing, Medical research

## Abstract

Biased and poorly documented dermatology datasets pose risks to the development of safe and generalizable artificial intelligence (AI) tools. We created a Dataset Nutrition Label (DNL) for multiple dermatology datasets to support transparent and responsible data use. The DNL offers a structured, digestible summary of key attributes, including metadata, limitations, and risks, enabling data users to better assess suitability and proactively address potential sources of bias in datasets.

## Introduction

With the growing adoption of artificial intelligence (AI) in a wide range of real-world applications, eXplainable artificial intelligence (XAI) has become a rapidly evolving area of research for responsible AI development^1,2^. XAI techniques aim to make complex models more interpretable and trustworthy so that human users can understand how decisions are made and determine when a model’s predictions can be appropriately trusted^3^. One component of XAI is data explainability, as the composition, quality, and representativeness of training data fundamentally shape model behavior^4^. Transparency at the dataset level is particularly important in high-stakes domains, such as healthcare, where the use of biased datasets in model development has serious implications for clinical decision making^5,6^.

In dermatology, AI has shown considerable promise in skin lesion classification and detection of skin cancers, with models achieving performance comparable to or even surpassing that of expert clinicians^7,8^. However, growing concerns have emerged regarding the quality and documentation of the datasets used to train these AI models^9,10^. Many dermatology datasets are curated from select patient populations, lack standardized metadata, and provide limited information about image acquisition protocols or selection criteria^9^. These issues are often underreported, making it difficult to evaluate dataset limitations and determine their suitability for specific applications^11–13^. Problematic, obscure data threaten the generalizability of AI models and introduce risks when applied in diverse clinical settings. For example, Daneshjou et al. found that state-of-the-art skin lesion classification models performed significantly worse on images of individuals with darker skin tones compared to those with lighter skin tones, likely due to underrepresentation of patients with darker skin in training data^14^. Such disparities underscore how opaque datasets can propagate bias in AI performance and ultimately undermine equitable care.

As the number of available datasets and models continues to rapidly grow, the need to critically evaluate dataset quality and fitness for use becomes increasingly important. Currently, researchers can find and access datasets through public repositories, institutional collaborations, literature mining, and proprietary agreements. However, there is no standardized reporting across these platforms to highlight dataset risks. Finding this information can be difficult or even impossible, limiting users’ ability to assess whether a dataset is appropriate for a given application^9^.

In response to these challenges, the Data Nutrition Project introduced the Dataset Nutrition Label (DNL), a structured dataset reporting framework introduced in 2018 to promote transparency and highlight important risks in datasets^15^. Drawing inspiration from food labeling, the DNL highlights the key “ingredients” in a dataset: metadata, representation, intended use cases, and known issues, all of which collectively determine whether a dataset is “healthy” for its intended applications^16^. The DNL is available as an easily accessed webpage and it enables users to audit datasets without needing direct access to the raw data, an important advantage given that data pre-processing is often time-consuming and typically requires domain expertise^17^. By providing a user-friendly overview of potential limitations of datasets, the DNL aims to support more responsible and equitable data practices in AI development. So far, the DNL has been applied to several datasets in domains beyond healthcare, such as the LAION-5B (“Large-scale AI Open Network”) image-text dataset which has supported the training of large-scale multimodal models (DNL can be accessed via: https://labelmaker.datanutrition.org/)^18^. Within dermatology, the DNL has also been previously applied to high-impact dermatologic datasets, such as the 2020 International Skin Imaging Collaboration (ISIC) dataset, which contains over 30,000 dermoscopic images and has led to the development of numerous skin lesion classification models (see Data Availability Statement)^19^. In this case study, we present the development of a DNL for the latest ISIC dermatologic image set, which comprises image crops extracted from 3D total body photography (3D TBP).

## The DNL

We applied the DNL framework to the 2024 SLICE-3D (“Skin Lesion Image Crops Extracted from 3D Total Body Photography”) Challenge dataset, a large, publicly available dataset comprising over 400,000 cropped lesion images derived from 3D TBP, an imaging technology that captures full-body photographic reconstructions to enable skin lesion tracking^20^. Collected from seven dermatology centers worldwide, the images in this dataset are intended to resemble smartphone-quality photos, as opposed to the high-resolution dermoscopic images featured in previous ISIC datasets^19,21,22^. The image type used in SLICE-3D is particularly relevant for applications in teledermatology and primary care. SLICE-3D was the foundation of the 2024 ISIC Challenge, which tasked participants with building models capable of distinguishing malignant from benign skin lesions^20^. The Challenge has engaged over 13,000 entrants and generated more than 79,000 submissions, illustrating the impact that a single dataset can have in sparking large-scale AI model development across a global research community.

**Fig. 1:**
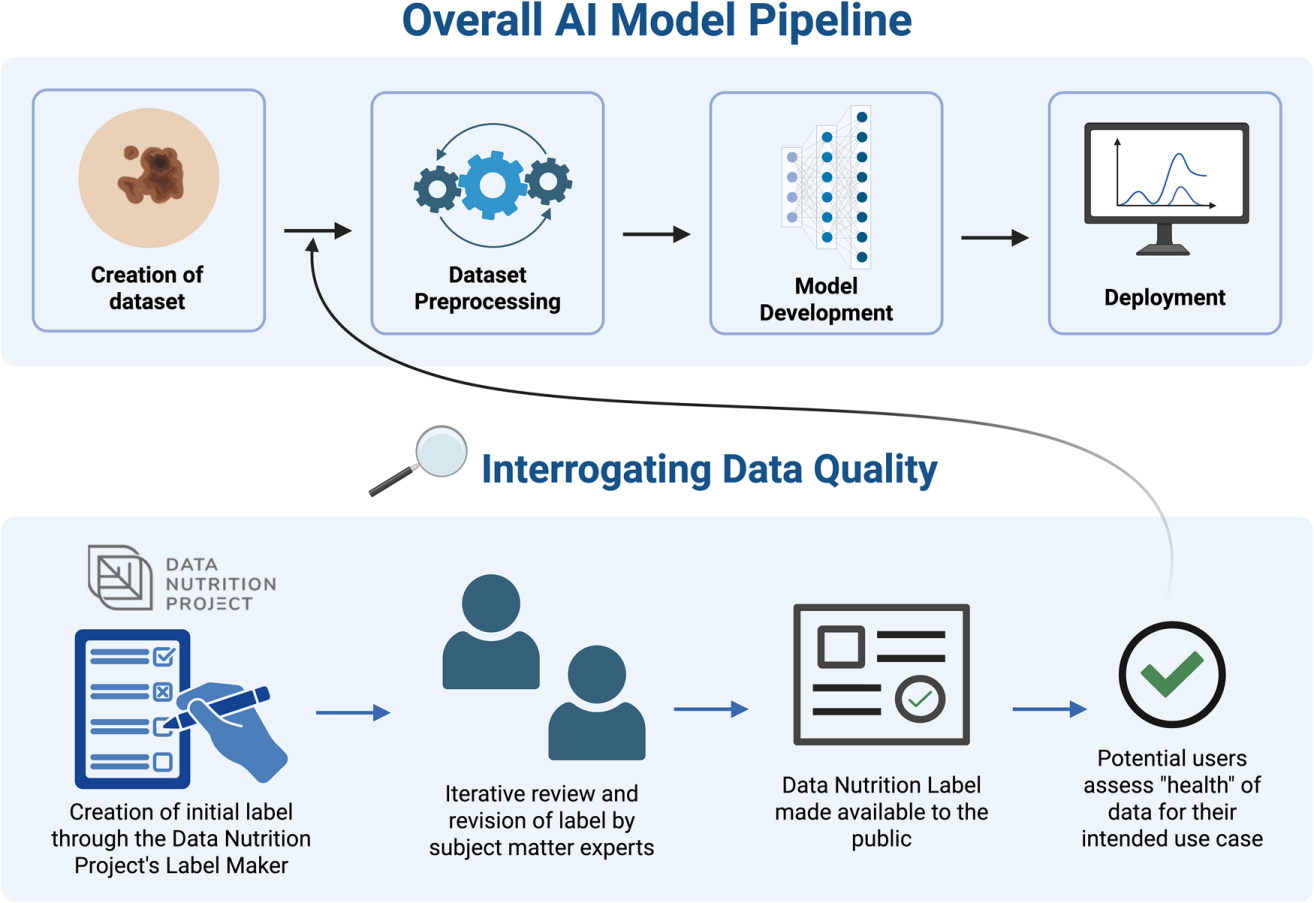
Schematic illustrating how the creation of a Data Nutrition Label (DNL) fits within the broader artificial intelligence (AI) model development pipeline. Created in BioRender: https://app.biorender.com/profile/template/details/t-68e174484289dc9ed5620e66-dnl-pipeline.

**Fig. 2:**
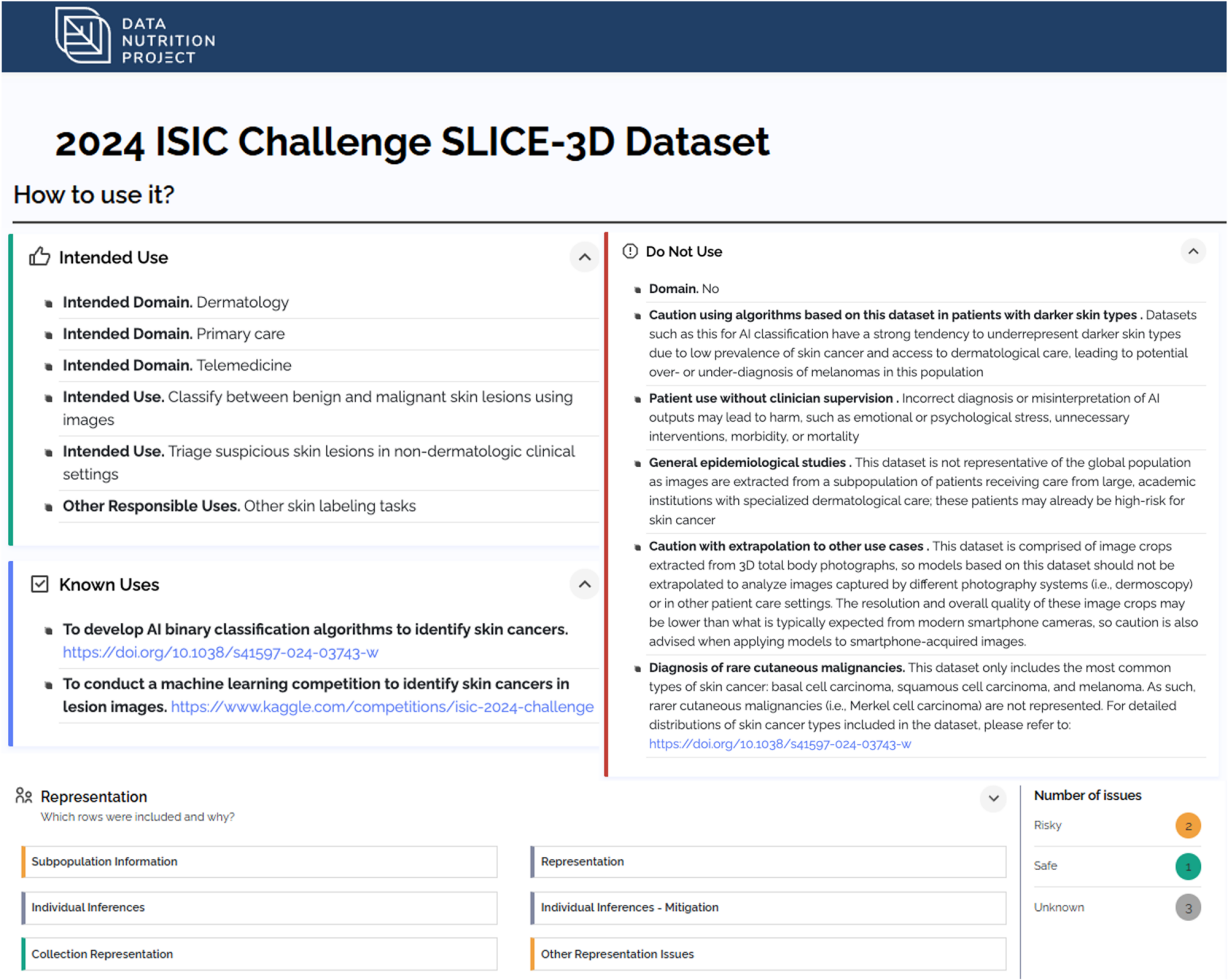
Interface display of the SLICE-3D Data Nutrition Label (DNL). A subset of components from the finalized DNL for the 2024 SLICE-3D ISIC Challenge Dataset is shown, including intended uses, prohibited use cases, and an example of the inference risk assessment. Inference risks are flagged using a color-coded severity scale to assist users in assessing the dataset’s limitations in context of their intended applications. Additional sections and justifications are available by expanding each item in the full label.

To construct the label, we used the Label Maker interface developed by the Data Nutrition Project. This interactive, web-based tool guides DNL creators through a series of approximately 60 standardized questions encompassing dataset ownership, licensing, data collection and annotation protocols, ethical review, intended use cases, and identified risks^15^. The total number of questions may vary slightly due to use of branching questions. Our responses were drawn from the SLICE-3D dataset descriptor, Kaggle-hosted metadata, and direct correspondence with dataset curators^20^. Two subject matter experts (SMEs) in dermatology and AI then reviewed the draft label for clinical and technical accuracy. Both SMEs were early- to mid-career, board-certified dermatologists and physician-scientists with extensive research experience in the clinical application of AI in dermatology. They participated in remote working sessions during which they reviewed the responses used to construct the DNL and provided feedback on clinically relevant considerations for datasets used to develop skin lesion classification models. The label was then revised iteratively based on their input. Once all SME feedback was fully incorporated, the final label was approved by the Data Nutrition Project team and made publicly available. Figure 1 illustrates where the DNL fits within the AI model development pipeline and how it contributes to responsible model creation. Figure 2 provides a view of the completed SLICE-3D DNL, illustrating how key dataset attributes, limitations, and risks are visually summarized for end users.

The DNL outlines technical details on data collection and deidentification, as well as flags limitations and risks associated with the dataset. Limitations specific to the SLICE-3D dataset include the absence of skin tone documentation, significant class imbalance between benign and malignant diagnoses, the exclusion of rare diagnoses, such as Merkel cell carcinoma, the low resolution of image crops even in comparison to modern smartphone photographs, and the potential for hidden proxies or underrepresented populations. Each potential risk was assigned a color-coded category to communicate its potential impact and level of concern: green for “safe”, yellow for “risky”, and gray for “unknown”. By alerting users to these risks, the DNL provides an opportunity to mitigate downstream harm by refining model design to account for these risks or avoiding using the dataset for model building altogether. The label also specifies appropriate use cases, such as training triage algorithms using non-dermoscopic images. It further cautions against less suitable applications, including diagnosing rare lesion subtypes or deploying models on individuals with darker skin tones.

## Discussion

The SLICE-3D DNL serves as an example of how standardized transparency tools may support responsible data practices in dermatology, a domain uniquely reliant on photographic data. Despite the increasing use of AI in dermatologic research, many datasets remain poorly characterized: they are often not publicly available, lack metadata, such as skin tone, and exhibit substantial class imbalance^9^. Many existing datasets are curated collections of lesion images selected for biopsy or monitoring, typically from academic centers, which introduces selection bias and limits generalizability. Given the central role of clinical photography in dermatology, issues regarding image quality, patient privacy, and representativeness are especially pertinent. As the SLICE-3D dataset remains publicly available for future model development, the accompanying DNL serves as a practical resource for assessing its continued relevance, limitations, and appropriate applications.

Compared to other transparency tools, such as audit frameworks, data statements, data descriptors, and datasheets, the DNL offers a more digestible format and leverages a standardized design for increased legibility^15,23,24^. By providing a streamlined summary focused on the most relevant considerations, it enables model developers and data scientists to directly compare datasets and assess fitness for use prior to model training. For example, DNLs emphasize a key distinction between the 2020 ISIC dataset, which includes high-quality dermoscopic images, and the 2024 SLICE-3D dataset, which consists of lower-resolution 3D TBP image crops of benign and malignant skin lesions. Since these datasets represent different image types, models trained on the 2020 dataset may be better suited for dermatologists who routinely use dermoscopy, while models trained on the 2024 dataset may be more useful in triage settings where images may be captured with smartphones, such as photographs submitted by patients. Summarizing both datasets using the same structured format allows users to make side-by-side comparisons across sections of the DNL so that they can efficiently evaluate risks and intended applications to guide responsible dataset selection.

To be effective, however, DNL creation still requires access to metadata, which may not always be available. Additional limitations include the need for manual input and expert review, both of which can be resource-intensive and may not be feasible. The process also involves a degree of subjectivity and may introduce variability across labels. Furthermore, the direct impact of the DNL on downstream dataset selection and model performance remains underexplored. In order to increase scalability and adoption, ongoing efforts aim to streamline DNL creation through automation and incorporation of quantitative summaries, such as data distribution visualizations^25^. Future research should emphasize real-world utilization of DNLs to assess their impact and refine its components.

Looking ahead, transparency tools like the DNL could be integrated into data collection, institutional review, or data governance workflows to promote best practices in dataset curation and documentation. In dermatology and other image-based specialties, structured labeling of datasets can support responsible model development while mitigating the risk of propagating bias. However, one must consider that standardized reporting is only a necessary starting point and that the DNL does not directly mitigate deep structural biases. Long-term solutions must also address underlying drivers of inequity. These include broadening datasets to include diverse populations and encouraging high-quality prospective data collection beyond those solely acquired from academic institutions^26,27^. Overall, we strongly encourage dataset curators to adopt structured labeling practices, such as the DNL, and to contribute to a broader ecosystem of responsible AI in dermatology.

## Data Availability

The DNL for the SLICE-3D dataset is available at: https://datanutrition.org/labels/v3/?id=6167d533-9d94-4afd-8e74-b3187175bc19. The DNL for the ISIC 2020 Challenge Dataset is available at: https://datanutrition.org/labels/v3/?id=93bd6f91-e224-4706-b463-bd5cbfb65e04. The full list of DNL questions and prompts is available here: https://docs.google.com/spreadsheets/d/1Wz9JCq66KKI7DJ4ElnpzYR3utqs9pBk5/edit?usp=sharing&ouid=102320259574749245861&rtpof=true&sd=true.
